# Microbiome profiling and Actinomycetes isolation from tropical marine sponges

**DOI:** 10.3934/microbiol.2025010

**Published:** 2025-03-03

**Authors:** Trinset Weeraphan, Chollabuppha Chou, Naphatson Chanthathamrongsiri, Thanchanok Sirirak, Sumaitt Putchakarn, Supakarn Chamni, Wongsakorn Phongsopitanun

**Affiliations:** 1 Department of Biochemistry and Microbiology, Chulalongkorn University, Pathum Wan, Bangkok, 10330, Thailand; 2 Faculty of Pharmaceutical Sciences, Burapha University, Chonburi, 20131, Thailand; 3 The Research Unit in Synthetic Compounds and Synthetic Analogues from Natural Product for Drug Discovery (RSND), Burapha University, Chonburi, 20131, Thailand; 4 Institute of Marine Science, Burapha University, 20131, Thailand; 5 Department of Pharmacognosy and Pharmaceutical Botany, Faculty of Pharmaceutical Sciences, Chulalongkorn University, Bangkok, 10330, Thailand; 6 Center of Excellence in Natural Products and Nanoparticles, Chulalongkorn University, Bangkok, Thailand

**Keywords:** marine sponge, microbiome, Actinomycetes, culture-independent techniques, culture-dependent techniques, bioactive compounds, 16S rRNA sequencing, bacterial diversity

## Abstract

Marine sponges are well-known for their production of bioactive compounds, many of which are synthesized by their associated symbiotic microorganisms. Among these, Actinomycetes are of particular interest due to their ability to produce secondary metabolites with antimicrobial and antitumor activities. We aimed to investigate the bacterial microbiome of tropical marine sponges, with an emphasis on the diversity and distribution of Actinomycetes, employing both culture-dependent and culture-independent approaches. Five sponge samples (PF01–PF05) were collected from Sichang Island, Chonburi Province, Thailand. The bacterial communities were analyzed using 16S rRNA gene sequencing and bioinformatics tools, revealing a significant microbial diversity dominated by Cyanobacteria, Actinomycetota, and Chloroflexi. Notably, PF01 (*Penares nux*) exhibited the highest microbial diversity, while PF05 (*Cacospongia* sp.) had the lowest. Actinomycetes, particularly the genus *Micromonospora*, were successfully isolated from all samples, with PF03 (*Ircinia mutans*) yielding the highest number of strains. Culture-independent analysis identified a greater proportion of unculturable Actinomycetes compared to those isolated through traditional methods, underscoring the limitations of culture-dependent techniques. This study enhances our understanding of sponge-associated microbial diversity and highlights the potential for isolating Actinomycetes from these sponges for novel drug discovery and other bioprospective applications.

## 1. Introduction

Marine sponges have received much attention in recent years due to their bioactive compounds, which offer promising prospects for the development of novel medications and healthcare innovations. Classified scientifically under the phylum Porifera, sponges constitute a diverse group with approximately 8,500 known species [1]. The phylum Porifera is divided into four classes based on skeletal composition: Calcerea, Demospongiae, Hexactinellida, and Sclerospongiae [2]. These multicellular aquatic animals inhabit primarily the ocean, exhibiting remarkable diversity in colors, shapes, and sizes. The unique characteristics of sponges are attributed to their bodies, which are filled with channels and pores that enable water to circulate through them, facilitating essential processes for nutrient absorption and providing a favorable environment for symbiotic relationships with microorganisms such as archaea, bacteria, cyanobacteria, and microalgae [3],[4]. Numerous secondary metabolites with potential biological activities have been identified in these symbiotic microorganisms within sponges [5],[6], such as bioactive peptides and polyketides [7], and antimicrobial compounds, such as avarol, aeroplysinin-1, preclathridine, spongiacidine B, and pyrrole alkaloids [8]. According to Dinarvand and Spain [9], marine extracts from the sponges were investigated to identify novel compounds with antimicrobial properties. The antibacterial screening of these extracts revealed that multiple compounds exhibited potent *in vitro* antibacterial activity, particularly against methicillin-resistant *Staphylococcus aureus* (MRSA).

Several studies indicate that these secondary metabolites are biosynthesized by the symbionts [10]–[12]. According to Skariyachan et al. [13], metabolites extracted from sponge-associated bacteria display inhibitory effects on pathogenic bacteria such as MRSA, *Proteus mirabilis, Klebsiella pneumoniae*, and *Salmonella typhi*. Sofyani et al. [14] reported that potential secondary metabolites with antimicrobial and antifungal properties are produced by sponge-associated bacteria. The synthesis of antimicrobial metabolites by these bacteria implies their potential role in protecting sponges against pathogens. One of the key symbiotic microorganisms identified in marine sponges for the biosynthesis of secondary metabolites is Actinomycetes [15]. The sponge-associated Actinomycetes are not only abundant and varied but also generate structurally novel secondary metabolites, including mainly polyketides [16], alkaloids [17], fatty acids, peptides, and terpenoids [18]. These compounds exhibit various biological activities, such as antibacterial, antitumor, and antiparasitic activities [19].

Actinomycetes are a group of Gram-positive to Gram-variable filamentous bacteria with high guanine + cytosine contents. Similar to fungi, they produce thread-like filaments identical to hyphae and form spore; however, their hyphae are generally smaller than fungal hyphae. They are exceptionally diverse, consisting of both benign and pathogenic bacteria [20]. Some species, such as *Mycobacterium tuberculosis* and *Nocardia* spp., can survive in harsh hospital environments and cause infectious diseases in humans [21], while others are tremendously high-impact sources of valuable bioactive compounds [22]. Species from the genus *Streptomyces*, for instance, produce a variety of secondary metabolites with high structural diversity [23], including streptomycin [24], clindamycin, chloramphenicol [25], fosfomycin, and ribostamycin [26].

Actinomycetes remain a valuable resource for the discovery of novel natural products, including antibiotics, due to their remarkable production of bioactive compounds and secondary metabolites [27],[28]. Unique environments, such as the marine environment, have provided access to strains capable of producing diverse antimicrobial compounds with varied chemical structures, including polyketides, nitrogen-containing compounds, sterols, and terpenoids [29]. Furthermore, a large number of antitumor compounds have been discovered [30]. Nevertheless, the increase in antimicrobial resistance has become a challenge in the development of new potential antibiotics [31]. Consequently, many researchers in recent years have shifted their focus to rare Actinomycetes or non-*Streptomyces* due to their ability to produce novel and effective antimicrobial compounds [32]. Many successful antibiotics, including macrolides [33], teicoplanin, and vancomycin [34], have been produced by rare Actinomycetes. The number of antibiotics produced by rare Actinomycetes has increased by 25–30% of known antibiotics in the last two decades [35]. Accordingly, rare Actinomycetes have become promising resources for the discovery of not only new antimicrobial compounds but also other bioactive metabolites with diverse properties.

Actinomycetes are present in various ecological habitats, including soils [36], freshwater [37], marine sediments [38], and marine sponges [39]. While microbiologists have traditionally concentrated on terrestrial environments due to the predominant presence of Actinomycetes in soils [40], recent investigations have revealed the potential of the marine environment as a distinct and promising source for rare Actinomycetes [41],[42]. The marine environment, especially marine sponges, provides a unique condition for rare Actinomycetes with distinct metabolic and genetic characteristics [43]. This diversity is driven by the extreme conditions prevalent in marine ecosystems, such as high salinity, pressure, and low temperatures [15]. Marine sponges are acknowledged as one of the most abundant sources of bioactive natural products, contributing to almost 30% of all-natural products identified from marine sources [44]. According to Sun et al. [45], marine-derived Actinomycetes were isolated from marine sponges and have been reported as a new source of aromatic polyketides. Many researchers have reported the isolation of rare Actinomycetes from marine sponges [46]. According to Olsen et al. [47], a novel Actinomycetes, *Tsukamurella spongiae*, was isolated from a deep-water marine sponge obtained from the coast of Curaçao in the Netherlands Antilles. In addition to sponges, marine Actinomycetes have been reported in symbiotic associations with corals and macroalgae. For instance, Actinomycetes associated with corals have been shown to produce compounds with unique antifungal and cytotoxic activities [48], while those associated with macroalgae have yielded metabolites with biotechnological potential, such as anti-inflammatory and antimicrobial agents [49]. These associations suggest that marine Actinomycetes adapted to challenging environments may be a rich source of novel biological resources [50]. Therefore, investigating marine Actinomycetes isolated from sponges, corals, and macroalgae may lead to the discovery of novel compounds with diverse bioactivities.

To study the communities of marine sponge-associated Actinomycetes, both culture-dependent and culture-independent methods were involved. These two methods can comprehensively evaluate the diversity and distribution of bacterial communities [51]. The conventional (culture-dependent) methods are laboratory procedures used to isolate and cultivate microorganisms. For centuries, the study of microorganisms relied heavily on the conventional and long-adopted culture-dependent method for investigating bacterial communities in different environments [52]. While these methods have historically facilitated the development of microbiology, their limitations are widely recognized and serious. [52]. Only a small portion of microorganisms from an environmental sample can be successfully cultured in the laboratory due to the selectivity of the nutrient media and culture conditions, resulting in the enormous bioprospecting potential of the uncultured diversity being overlooked [53].

In recent decades, culture-independent methods have been developed since the application of molecular methods [54]. Culture-independent methods do not rely on cultivation; instead, they target nucleic acids to investigate microorganisms in a particular ecosystem [55]. The application of molecular techniques in culture-independent methods makes them more accurate and appropriate for identifying microorganisms [56]. Next-generation sequencing (NGS), or large-scale massively parallel sequencing, has been developed [57]. NGS, such as Illumina sequencing, can sequence millions of DNA fragments simultaneously, offering comprehensive details on genome structure, genome variations, gene activity, and changes in gene behavior [58]. With its high-throughput capacity and cost-effectiveness, NGS has been applied and opened new opportunities for understanding microbial diversity [59].

In this study, we focused on sponge samples collected from Sichang Island in the Gulf of Thailand, a region with diverse marine ecosystems and unique environmental gradients influenced by industrial and conservation activities. Despite its ecological significance, the Gulf of Thailand remains underexplored for microbial diversity and natural product discovery, offering an opportunity to uncover novel Actinomycetes and bioactive metabolites. Consequently, we aim to isolate Actinomycetes from marine sponges in this region and investigate their biodiversity using culture-dependent and culture-independent methods. Metagenomic sequencing through 16S ribosomal RNA, such as Illumina MiSeq sequencing, was performed to evaluate the diversity of Actinomycetes and compare it to culture-dependent methods. Integrating these two methodologies enables a more thorough understanding of Actinomycetes communities in marine sponges.

## 2. Materials and methods

### 2.1. Sample collection and preparation

Five sponge samples, each weighing approximately 20–30 g (wet weight), were collected by scuba diving at depths of 3–5 meters near Sichang Island in the Gulf of Thailand. The collection was conducted with the assistance of the Aquatic Resources Research Institute, Chulalongkorn University, and under authorization from the Department of Fisheries, Ministry of Agriculture and Cooperatives, Thailand (Permit No. 0510.2/8234, dated October 28, 2019). Briefly, sponge samples were collected and immediately stored in an ice box to maintain a low temperature during transportation. Upon reaching land, the samples were transferred to −20 °C for storage until further processing. The sponges were not washed to retain their natural microbiome composition. Sponge samples were identified based on morphological and ecological features such as growth form, color, depth, and substrate. Details of collected sponges are provided in Table 1, Figures S1 and S2.

**Table 1.** List of sponge samples used in this study.

Number	Sample code	Sponge scientific name
1	PF01	*Penares nux* (de Laubenfels, 1954)
2	PF02	*Cacospongia* sp.
3	PF03	*Ircinia mutans* (Wilson, 1925)
4	PF04	*Gelliodes petrosioides* Dendy, 1905
5	PF05	*Cacospongia* sp.

### 2.2. DNA extraction and metagenomics sequencing for the bacterial microbiome in sponges

For metagenomic analysis, a small fragment was taken from each individual sponge sample for DNA extraction. DNA extraction from sponges was conducted utilizing the DNeasy PowerSoil Pro DNA Kit (Qiagen, USA). For library preparation, the amplification of V3-V4 region on the 16S rRNA gene was achieved using 2X sparQ HiFi PCR master Mix (QuantaBio, USA) in conjunction with 341F (5′-TCGTCGGCAGCGTCAGATGTGTATAAGAGACAGCCTACGGGNGGCWGCAG-3′) and 805R primers (5′-GTCTCGTGGGCTCGGAGATGTGTATAAGAGACAGGACTACHVGGGTATCTAATCC-3′), whereby the underlined sequences represent overhanging adaptors subsequently trimmed off. Thermocycling reactions proceeded as follows: Initial denaturation at 98 °C for 2 min followed by 30 cycles of denaturation at 98 °C for 20 s, annealing at 60 °C for 30 s, extension at 72 °C for 1 min, and a final extension step at 72 °C for 1 min. After PCR, the products were purified utilizing sparQ Puremag Beads (QuantaBio, USA) and then indexed using Nextera XT index primer (5 µL per 50 µL PCR reaction) through 8–10 cycles of PCR under identical conditions as mentioned earlier. The resulting PCR products were pooled and diluted to achieve a loading concentration of 4 pM. Cluster generation of DNA fragments and paired-end sequencing were performed at the Omics Sciences and Bioinformatics Center, Chulalongkorn University, Bangkok, Thailand, utilizing the Illumina MiSeq platform. The paired-end sequence data generated in this study were deposited in the NCBI Bioproject database under the accession number PRJNA1223106 and are publicly available for verification and further analysis.

### 2.3. Bioinformatics and diversity analysis

Bacterial microbiome informatics analysis was conducted using QIIME 2 version 2020.8, as described by Bolyen Rideout Dillon et al. [60]. Initially, raw sequence data underwent demultiplexing and quality filtering employing the q2-demux plugin, followed by denoising via DADA2 utilizing the q2-dada2 plugin [61]. Phylogenetic tree construction was facilitated by the SEPP q2-plugin, which placed short sequences into sepp-refs-gg-13-8.qza as a reference for phylogenetic tree generation [62]. Subsequently, alpha-diversity analysis, assessing diversity within samples, was performed utilizing Faith's Phylogenetic Diversity [63] and the Shannon metric [64] based on available operational taxonomic units (OTUs). Beta-diversity analysis, encompassing weighted and unweighted UniFrac distance metrics [65],[66] Jaccard distance, Bray-Curtis dissimilarity, and Principal Coordinate Analysis (PCoA), was conducted using q2-diversity. Taxonomy assignment to amplicon sequence variants (ASVs) was accomplished through the q2-feature-classifier using the classify-sklearn Naïve Bayes method against the Silva 13_8 99% OTUs reference sequences [67]. Taxonomic classification in this study was conducted using the Silva database, which references the phylum as Actinobacteriota. However, as this phylum has been reclassified as Actinomycetota [68], we have adopted the updated nomenclature in this manuscript while acknowledging that some databases and analysis tools may still use the former term. Visualization of OTUs data of Actinomycetes was performed by CCmetagen via Krona chart [69]. The diversity of Actinomycetes was analyzed using classified OTU data derived from 16S metagenomic sequencing. To quantify the diversity within the class Actinomycetes across various samples, the Shannon diversity index (*H′*) and Simpson's diversity index (*D*) were employed [70].

The Shannon diversity index was calculated from this formula

$$H^{\prime }=-\overset{s}{\underset{i=1}{\sum }}P_{i}(lnP_{i})$$

The Simpson's diversity index was calculated from this formula

$$D=1-\overset{s}{\underset{i=1}{\sum }}{\left(P_{i}\right)}^{2}$$

Where *H′* = the Shannon index value

  *D* = the Simpson's diversity index

  *P_i_* = the proportion of the population represented by the *i*-th species (or OTUs in this context)

  *S* = the total number of species (or total number of OTUs)

### 2.4. Isolation of Actinomycetes by a cultural method

One gram of sponge samples was aseptically ground and suspended in natural seawater collected directly from the sampling site. Serial dilutions (10^0^ to 10^−3^) were prepared using the same natural seawater as the diluent. A volume of 0.1 mL of the suspension was spread onto M2 (6 mL glycerol, 1 g arginine, 1 g K_2_HPO_4_, 0.5 g MgSO_4_, 15 g agar, 1 L seawater, pH 7.0) [71] and starch casein nitrate (SCN) medium (10 g soluble starch, 2 g K_2_HPO_4_, 2 g KNO_3_, 0.3 g sodium caseinate, 0.05 g MgSO_4_.7H_2_O, 0.02 g CaCO_3_, 0.01 g FeSO_4_.7H_2_O, 15 g agar, 1 L sea water, pH 7.0) [72], which contained 25 mg/L nalixidic acid, 50 mg/L cycloheximide, and 1 mg/L terbinafine. The agar plates were incubated at room temperature (approximately 25–28 °C) in dark conditions for a duration of 14 days. Following the incubation period, Actinomycetes colonies were observed to have grown, and they were purified using the streak plate technique. The purified colonies were then stored on International *Streptomyces* Project-2 (ISP2) agar medium (4 g yeast extract, 10 g malt extract, 4 g glucose, and 20 g agar per liter of seawater) for further study and analysis. For long-term storage, the isolates were maintained as glycerol stocks (20% glycerol) at -80 °C.

### 2.5. Identification of isolated Actinomycetes

The isolates of Actinomycetes were cultured in ISP2 broth on a rotary shaker (200 rpm) at 30 °C for seven days. Subsequently, genomic DNA was extracted using the PureLink™ Genomic DNA Mini Kit (Thermo Fisher Scientific, USA). Amplification of the 16S rRNA gene was conducted through the polymerase chain reaction (PCR) technique employing primers 20F (5′-GAGTTTGATCCTGGCTCAG-3′) and 1500R (5′-GTTACCTTGTTACGACTT-3′) as described by Suriyachadkun et al. (2009). The PCR protocol included an initial denaturation at 94 °C for three minutes followed by 30 cycles of 94 °C for one minute, 50 °C for two minutes, and 72 °C for two minutes, culminating in a final extension at 72 °C for seven minutes. After amplification, the PCR products were purified using the GenepHlow™ PCR Cleanup Kit (Geneaid, Taiwan), thereby preparing the amplicons for subsequent sequencing. Sequencing was performed using universal primers by Macrogen, Korea, a service that ensures high fidelity in sequence data production. The resultant 16S rRNA gene sequences were aligned using the BioEdit Sequence Alignment Editor software [73]. Comparative analysis was then conducted utilizing EzBioCloud [74]. The DNA sequence data of isolated Actinomycetes in this study were deposited in the National Center for Biotechnology Information (NCBI) database (https://www.ncbi.nlm.nih.gov/). The assigned accession numbers are provided in the supplementary information (Table S1).

## 3. Results

### 3.1. Bacterial microbiomes found in sponges

To investigate bacterial communities in marine sponges, 16S metagenomic sequencing was employed in five sponge samples (PF01 to PF05). OTUs were clustered and assigned. Overall, 132,805 OTUs were observed and classified as 284 unique OTUs data. The top five phyla found across samples were Cyanobacteria 41,903 OTUs (31.55%), Actinobacteriota (Actinomycetota from recent reclassification) 19,515 OTUs (14.69%), Chloroflexi 16,389 OTUs (12.34%), Proteobacteria 11,429 OTUs (8.61%), and Acidobacteriota 11,071 OTUs (8.34%). On the family level, the unidentified family within Synechococcales (Cyanobacteria) accounted for the largest proportion of OTUs across all samples, with a total of 39,267 OTUs. Additionally, *Microtrichaceae* (Actinomycetota, 11,796 OTUs), *Rhodothermaceae* (Bacteroidota, 8,389 OTUs), and an unidentified family within Thermoanaerobaculales (Acidobacteriota, 8,170 OTUs) also contributed significantly to the bacterial diversity (Table S2).

**Figure 1.**
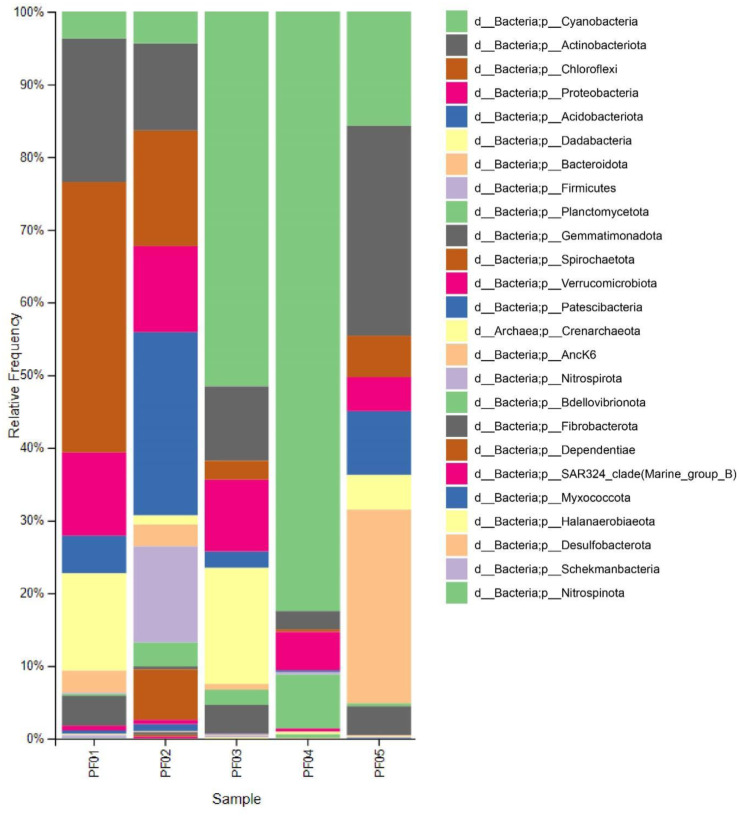
Taxonomic composition of the bacterial microbiome in sponge samples at the phylum level. PF01–PF05 represent individual sponge samples analyzed in this study. The relative frequency of bacterial phyla is shown for each sample, highlighting the microbial community composition. PF01, *Penares nux*; PF02, *Cacospongia* sp.; PF03, *Ircinia mutans*; PF04, *Gelliodes petrosioides*; and PF05, *Cacospongia* sp.

Sample-specific trends emerged from the data. PF01 exhibited the highest levels of Chloroflexi, while PF04 had the greatest abundance of Cyanobacteria. Actinomycetota was more prevalent in PF05 compared to the other samples. An inverse relationship between the abundance of Cyanobacteria and Chloroflexi was observed across the sponge samples. For instance, Cyanobacteria were highly abundant in PF04, while Chloroflexi were less prevalent. Conversely, PF01, which had the highest levels of Chloroflexi, exhibited lower Cyanobacteria abundance (Figure 1).

### 3.2. Diversity analysis of bacterial microbiomes

Comprehensive microbial community profiling across five sponge samples (PF01 to PF05) revealed diverse taxonomic landscapes, with significant variations in both richness and phylogenetic breadth. Alpha diversity assessments, including rarefaction analyses and diversity indices, indicated that PF01 harbored the most diverse microbiota. The Shannon diversity index demonstrated high species richness and a favorable balance between species abundance and evenness, supported by a steep initial slope in the rarefaction curve that plateaued at increased sequencing depths, signaling a saturation of diversity. Faith's Phylogenetic Diversity further confirmed that PF01 contained a wide range of phylogenetic lineages, indicating the presence of both common and rare taxa with extensive evolutionary histories.

**Figure 2.**
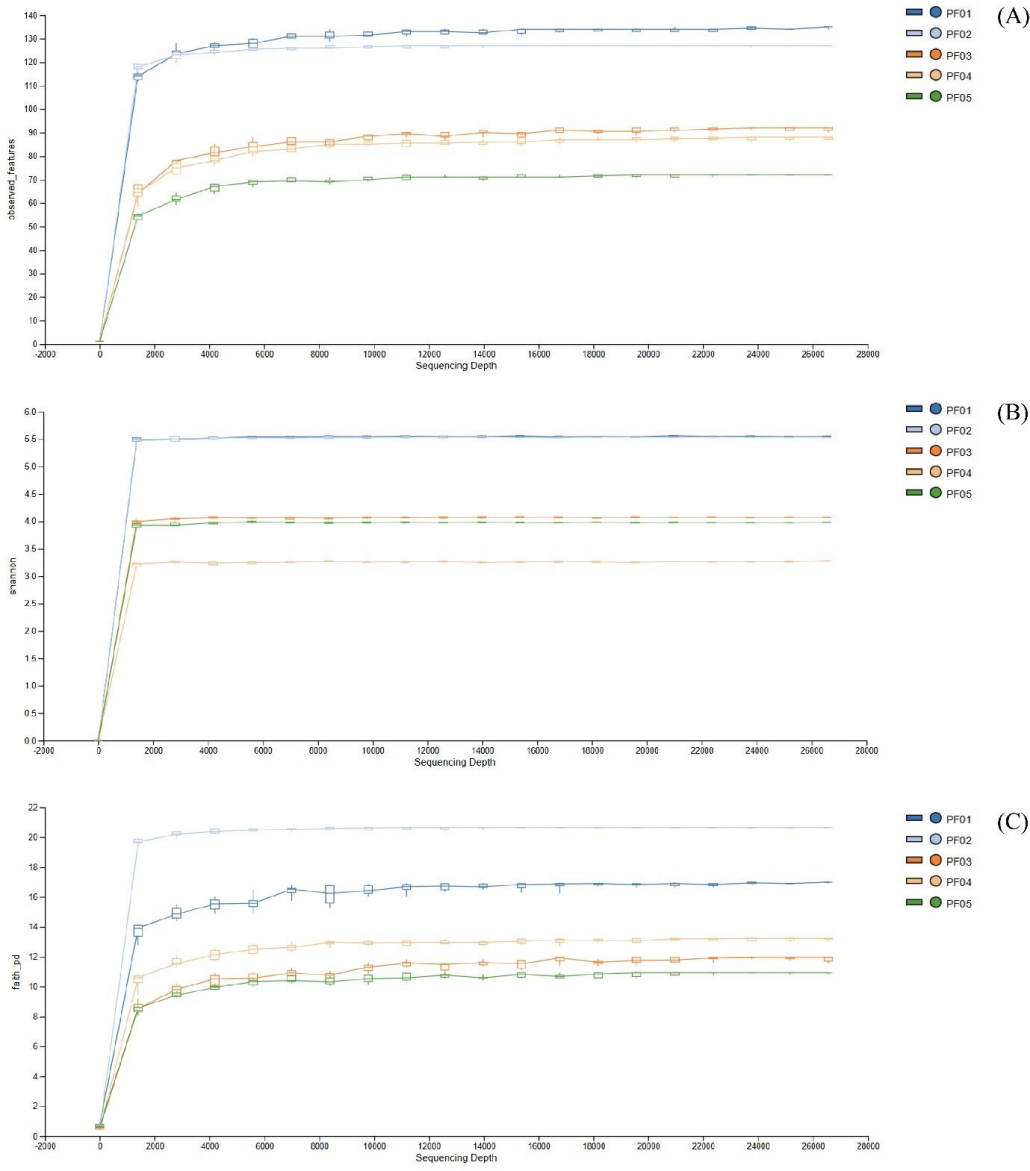
Rarefaction curves of the bacterial microbiome in sponge samples, representing diversity metrics. (A) Observed features curve, (B) Shannon diversity curve, and (C) Faith's phylogenetic diversity curve.

In contrast, PF05 exhibited the lowest alpha diversity metrics, with a rarefaction curve that rapidly plateaued. This indicated a limited range of species, corroborated by consistently lower values in both the Shannon index and observed OTUs, suggesting a community structure that is less rich and less even. Samples PF02, PF03, and PF04 displayed intermediate and comparable levels of microbial diversity, with their rarefaction curves and diversity metrics showing similar patterns (Figure 2).

**Figure 3.**
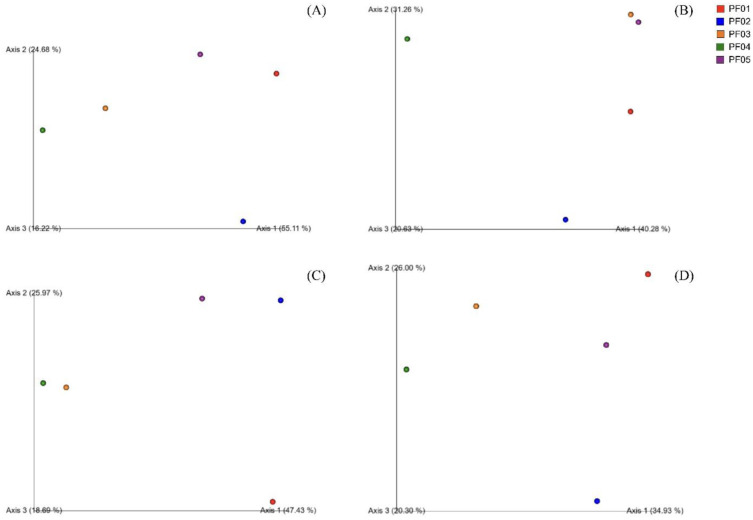
Principal Coordinate Analysis (PCoA) plots of beta diversity metrics for the bacterial microbiome in sponge samples. (A) weighted UniFrac PCoA, (B) unweighted UniFrac PCoA, (C) Bray-Curtis PCoA, and (D) Jaccard PCoA.

Beta diversity analyses, using weighted and unweighted UniFrac, Bray-Curtis, and Jaccard in Principal Coordinate Analysis (PCoA), provided insights into the microbial community structure and composition across samples. Weighted UniFrac and Bray-Curtis metrics, which account for the relative abundance of taxa, identified PF01 as having a unique microbial composition. PF02, PF03, and PF05 showed varying degrees of similarity in their microbial community structures, whereas PF04 exhibited unique microbial taxa not found in other samples, as highlighted by unweighted UniFrac and Jaccard analyses (Figure 3).

### 3.3. Diversity analysis of Actinomycetes communities

The analysis of OTU data for the class Actinomycetes across the five sponge samples (PF01, PF02, PF03, PF04, and PF05) provided detailed insights into their abundance and diversity. In terms of total abundance, PF04 contained the highest number of Actinomycetes OTUs (434), followed by PF03 (303 OTUs), PF05 (125 OTUs), PF02 (99 OTUs), and PF01 (53 OTUs) (Table S2).

**Figure 4.**
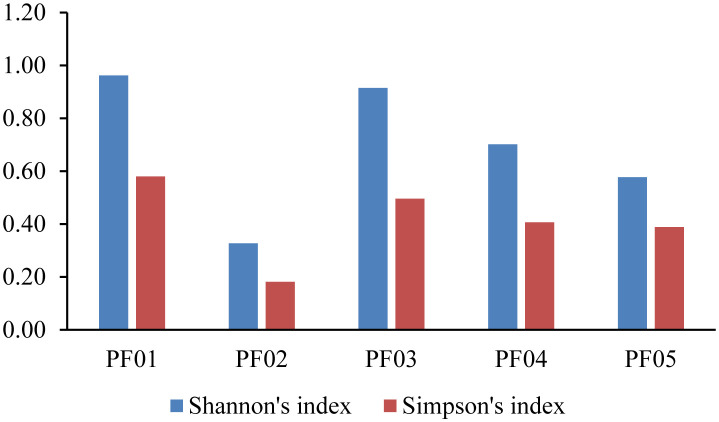
Comparison of Shannon's and Simpson's diversity index based on Actinomycetes OTUs in sponge samples PF01 through PF05.

Diversity indices, including the Shannon and Simpson indices, were calculated to assess the community structure across the samples. The Shannon diversity index was highest in PF01 (0.96), reflecting a highly diverse and evenly distributed Actinomycetes community, including genera such as *Bifidobacterium* and *Micromonospora*. In contrast, PF02 showed the lowest Shannon value (0.33), indicating a less diverse community dominated by a few species, primarily unculturable Actinomycetes. Intermediate values were observed in PF03 (Shannon: 0.91, Simpson: 0.50), PF04 (Shannon: 0.70, Simpson: 0.41), and PF05 (Shannon: 0.58, Simpson: 0.39), reflecting varying levels of diversity and species dominance across these samples. The Simpson index, which emphasizes species dominance within a community, supported these findings. PF01 had the lowest Simpson value (0.58), indicating less species dominance, while PF02 had the highest value (0.18), suggesting strong dominance by one or a few species (Figure 4).

### 3.4. Isolation of culturable Actinomycetes from sponges

In this study, Actinomycetes were successfully isolated from sponge samples PF01–PF05, representing a diverse range of species. Two media, M2 and starch casein nitrate (SCN), were employed to isolate Actinomycetes, resulting in the recovery of 48 strains. PF03 yielded the highest number of isolates (19 strains), followed by PF02 (15 strains), PF05 (6 strains), PF04 (5 strains), and PF01 (3 strains) (Table S1).

Most isolated strains belonged to the genus *Micromonospora* (45 strains), while the remaining three strains were identified as *Streptomyces*. *Micromonospora* was dominant in all sponge samples based on culture-dependent methods. However, the microbiome data detected *Micromonospora* only in PF01. This discrepancy suggests that *Micromonospora* strains present in other sponge samples may be at low abundance, potentially escaping detection via sequencing methods [75],[76]. Additionally, SCN medium may have favored the growth of *Micromonospora* over other taxa, as previous studies have demonstrated its efficacy in yielding high colony-forming units from environmental samples [77],[78]. Among the isolated *Micromonospora* strains, the most frequently identified species were *Micromonospora fluminis* (11 isolates), *Micromonospora maritima* (6 isolates), and *Micromonospora schwarzwaldensis* (5 isolates).

**Figure 5.**
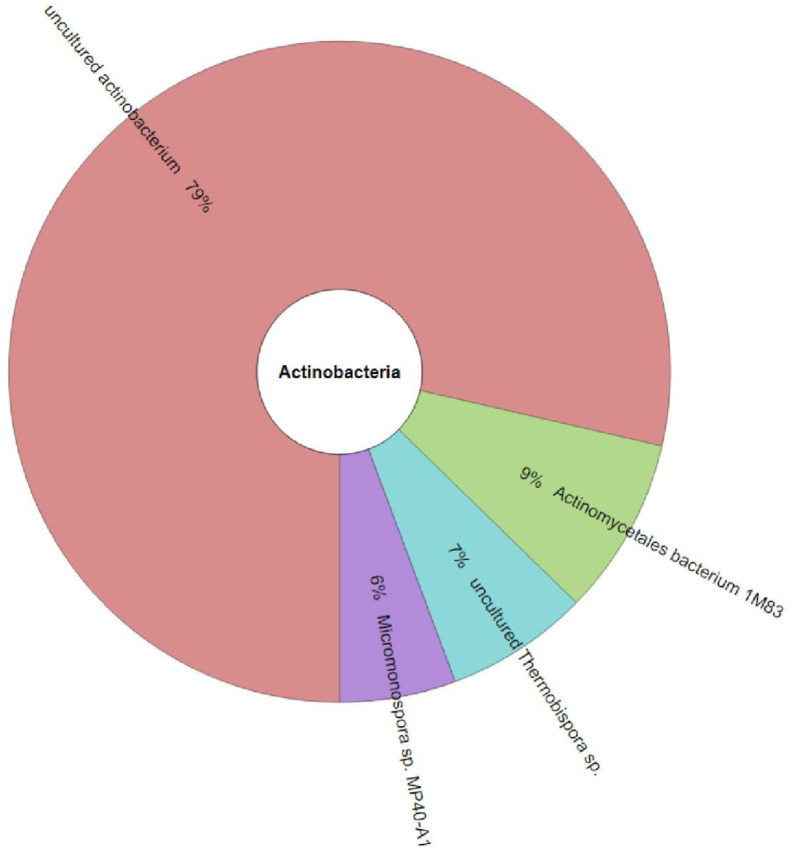
Krona chart visualization of the combined community of Actinomycetes composition observed in the microbiome data for all five sponge samples analyzed using CCmetagen.

The isolation of Actinomycetes yielded only *Streptomyces* and *Micromonospora* species, which reflects a subset of the diversity detected in the NGS data. The cross-validation performed using CCmetagen analysis revealed that more than 80% of Actinomycetes identified from the sponge samples were classified as unculturable (Figure 5), highlighting the complementary nature of culture-based and sequencing approaches. This suggested that the culture-based methods capture only a fraction of the total Actinomycetes diversity present in sponge-associated microbiomes.

## 4. Discussion

In this study, several marine sponge species were collected and identified from Sichang Island, Chonburi Province. The distribution pattern of these sponges was reported along the Gulf of Thailand (Chonburi and Rayong Province). *Penares nux* (PF01) and *Cacospongia* sp. (PF02 and PF05) were observed in both industrial and conservation areas, specifically in the Sichang and Mun Islands, indicating their resilience to varying environmental conditions. This reflects their ecological flexibility and ability to thrive in diverse marine environments. The widespread presence of these sponges suggests their potential as a bioindicator, particularly for ecosystems exposed to anthropogenic stressors. Furthermore, *Ircinia mutans* (PF03) was primarily found in industrial areas, particularly in regions characterized by high sedimentation, such as reef slopes and soft-bottom habitats. Its abundance in these areas suggests that *I. mutans* could be an indicator species for assessing sedimentation levels in marine ecosystems. *Gelliodes petrosioides* (PF04) was also recorded in both industrial and conservation zones, with a notable presence in coral reef habitats. The distribution of this species across environmental gradients points to its adaptability and ecological significance in nutrient cycling within coral reef ecosystems [79]. In this study, we focused on microbial diversity in sponge-associated communities, but whether sponge adaptability to varying environments influences microbiome diversity remains unclear. Future studies integrating microbiome analysis with environmental and ecological data could explore this relationship.

The findings from sponge distribution align with this research objective for exploring the bacterial microbiome associated with marine sponges. The diverse sponge species identified across locations, including industrial and conservation zones, provide important context for the microbiome profiles observed in each sponge. The dominance of Cyanobacteria, particularly the unidentified family within Synechococcales, suggests that these bacteria play a significant role in the sponge microbiome. Cyanobacteria are well-known for forming symbiotic relationships with sponges, offering mutual benefits. The sponges provide a protected habitat and access to nutrients, while Cyanobacteria contribute to sponge nutrition through photosynthesis and nitrogen fixation. Nitrogen fixation is particularly important in nutrient-poor marine ecosystems, where it supports not only sponges but also other organisms like corals [80]. Along with the Gulf of Thailand, PF01 was found in both industrial and conservation areas [79] and exhibited the highest microbial diversity in this study, with Cyanobacteria being dominant. This could be linked to the varied environmental conditions of its habitat, where the sponge is exposed to both nutrient-rich and anthropogenically influenced waters.

The inverse relationship between Cyanobacteria and Chloroflexi abundance suggests potential ecological interactions or competition between these two phyla. Chloroflexi, which are photoheterotrophs relying on organic compounds for carbon, may be limited in environments where Cyanobacteria dominate, as Cyanobacteria perform oxygenic photosynthesis, potentially monopolizing light in shallow water environments. This competition may restrict Chloroflexi's access to light-dependent energy sources, limiting their growth [81]. Additionally, sponges may exert selective pressures to favor their symbionts, using chemical defenses to mitigate competition from other microbes, including Chloroflexi [82].

Actinomycetota, particularly members of the class Acidimicrobiia, play crucial roles in degrading complex organic compounds, including aromatic compounds and pollutants like polycyclic aromatic hydrocarbons (PAHs) [83],[84]. The variable abundance of Actinomycetota across the samples likely reflects differences in the ecological roles they perform, particularly in organic matter degradation and nutrient cycling. The high abundance of Actinomycetota in PF05 and PF01 could indicate active involvement in nutrient cycling, supporting the metabolic needs of the sponge by transforming recalcitrant organic molecules into usable forms. The lower abundance of Actinomycetota in PF04 may reflect a reduced reliance on these microbial functions, potentially due to differences in nutrient availability or microbial competition.

Proteobacteria and Acidobacteriota were consistent contributors across all samples. The Gammaproteobacteria class, particularly Steroidobacterales, is known for its ability to degrade steroids via the aerobic 9,10-seco steroid degradation pathway. The presence of steroid-degrading bacteria in sponges may suggest a symbiotic relationship where these bacteria assist in breaking down steroids, preventing harmful accumulation within the sponge [85]. However, the lower abundance of Proteobacteria in PF05 may indicate competition with other bacterial groups like Cyanobacteria, which dominate in this sample.

Thermoanaerobaculia (Acidobacteriota) displayed variable abundance across samples, with PF02 and PF05 showing higher levels than other samples. The prevalence of Thermoanaerobaculia in PF02 suggests that these bacteria might play a role in the sponge microbiome under low-oxygen or high-temperature conditions, as this class is known for thriving in such environments [86]. Their ability to adapt to thermal stress could be crucial for the sponge's resilience in changing marine environments [87]. Additionally, Vicinamibacteria, though recently discovered and poorly understood, were found distributed across all samples, indicating their potential involvement in sponge holobionts, although their specific roles require further investigation [88].

Beta diversity analyses revealed unique microbial compositions across the samples, with PF01 showing a particularly distinct microbial community. This uniqueness may be influenced by environmental conditions or specific traits of the host sponge. For instance, PF01 has been associated with the production of bioactive compounds like trisoxazole macrolides, known for their antimicrobial and antifouling properties [89]. The uneven distribution of these compounds within the sponge, such as the concentration of kabiramides in certain structures like the capitums, may lead to spatial variations in microbial communities, as different bacterial species respond to the presence of these bioactive molecules. Additionally, the fatty acid profile of *Penares* species, particularly chloro-derivatives, may further influence microbial associations by interacting with microbial metabolites [90]. PF04 also exhibited a unique microbial community, suggesting niche specialization or geographical isolation that contributes to the presence of distinct microbial taxa. The similarity between PF03 and PF05 in beta diversity metrics suggests that these sponges experience similar environmental conditions or host-specific traits, which may influence the selection of comparable microbial taxa. These shared microbial interactions could play crucial roles in ecological resilience, enabling these sponge-associated communities to adapt to environmental stressors.

The observed patterns of Actinomycetes abundance and diversity highlight the differing ecological conditions and microbial interactions across the sponge samples. The high Shannon index in PF01 suggests a stable environment with minimal competition, promoting an evenly distributed and diverse Actinomycetes community. This sample included a variety of taxa, such as *Bifidobacterium* and *Micromonospora*, indicating that these genera might thrive in environments that support balanced microbial interactions. Such conditions are conducive to the formation of complex symbiotic relationships within the sponge microbiome, potentially contributing to its health and function.

Conversely, the low Shannon index in PF02 suggests a microbial community under environmental stress or constrained by limited ecological niches, which favors a few dominant species. The high Simpson index in PF02 further indicates that one or a few species are predominant, reducing overall diversity. These conditions might result from factors such as nutrient limitations, competitive exclusion, or other environmental stressors that limit the proliferation of a diverse microbial community. PF03, PF04, and PF05 displayed intermediate levels of Actinomycetes diversity, with PF03 showing a richer Actinomycetes community, including uncultured marine *Propionibacteriaceae*. This suggests that PF03 may be experiencing different ecological interactions compared to PF04 and PF05, where the presence of similar taxa in different proportions points to varying environmental pressures or nutrient availability. The balance of species dominance in these samples, as reflected by the Simpson index, suggests that microbial interactions are more dynamic in these environments, enabling a more even distribution of species.

The isolation of Actinomycetes in this study showed that *Micromonospora* was the dominant genus under the conditions employed, which may point to its competitive advantage within the sponge microbiome. However, it is important to note that different sampling approaches, storage conditions, or the use of diverse isolation media could potentially result in the isolation of a broader range of Actinomycetes. Studies have shown that *Micromonospora* strains can be isolated from various marine environments, including deep-sea sponges, where they produce bioactive compounds with notable antibiotic properties [91],[92]. The ability of *Micromonospora* to produce inhibitory metabolites likely contributes to its dominance, enabling these strains to compete effectively for space and resources within the sponge environment. Moreover, the discrepancy between NGS data and culturing results suggests that *Micromonospora* strains, while present in low abundance in some samples, can be selectively cultured under specific laboratory conditions. The use of SCN medium likely favored the growth of *Micromonospora*, supporting previous findings that certain media can enhance the isolation of Actinomycetes from environmental samples [77]. This highlights the limitations of both culture-based methods, which capture only a portion of microbial diversity, and sequencing approaches, which may overlook low-abundance taxa. Additionally, a limitation of this study is the use of only M2 and SCN media for Actinomycetes isolation. While effective, these media may not support the growth of all Actinomycetes present in the samples, potentially restricting the diversity of strains isolated. Future studies could incorporate additional media formulations to capture a broader range of Actinomycetes and provide a more comprehensive representation of the Actinomycetes community.

In addition, this discrepancy highlights the limitations of the universal primers (341F/805R) used in this study for 16S rRNA sequencing, as they may not sufficiently capture the diversity of certain taxa like Actinomycetes. While these primers are effective for broad-spectrum bacterial profiling, they can underrepresent low-abundance taxa or those with mismatched primer binding sites. Actinomycetes-specific primers, such as those suggested by previous studies (e.g., ACT1360R/ACT283F, F243/R513), could provide improved resolution of Actinomycetes diversity. Future studies incorporating such primers could further elucidate the contributions of this important bacterial group to sponge microbiomes. Additionally, the storage of samples at -20 °C before processing, while ideal for preserving microbial DNA for microbiome-based studies and preventing contamination during transport and storage, may negatively impact the survival of non-spore-forming Actinomycetes. This could result in an underestimation of their diversity in culture-dependent methods.

The diversity of isolated *Micromonospora* species, including *M. fluminis*, *M. maritima*, and *M. schwarzwaldensis*, reflects their adaptability to various environmental conditions. For example, *M. maritima* has been reported to thrive in stressful environments, such as mangrove soils, which are characterized by high salinity and osmotic pressure, suggesting that these species possess enzymes and metabolites that enable them to break down complex compounds and contribute to nutrient cycling within their habitats [93]. The pharmaceutical potential of *Micromonospora* is particularly noteworthy, as they are known for producing a wide range of bioactive secondary metabolites. These include antimicrobial compounds effective against multidrug-resistant pathogens, positioning *Micromonospora* as a valuable resource for drug discovery [94]. Beyond pharmaceuticals, *Micromonospora* have significant biotechnological applications, particularly in industries requiring robust enzymes for processes such as food processing, textiles, and pharmaceuticals. The enzymatic capabilities of *M. maritima*, which thrives in harsh environments like mangrove soils, suggest its enzymes can be effective biocatalysts for degrading tough substrates in industrial applications [95].

The Actinomycetes detected and isolated in this study, particularly *Micromonospora* and *Streptomyces*, are consistent with previous reports of sponge-associated Actinomycetes. For example, *Micromonospora* species have been isolated from tropical marine sponges in the South China Sea and Indian Ocean, where they have been recognized for producing bioactive secondary metabolites [96],[97]. However, to our knowledge, these bacteria have not been reported from the specific sponge species sampled in this study (*Penares nux*, *Cacospongia* sp., *Ircinia mutans*, and *Gelliodes petrosioides*) in the Gulf of Thailand. This suggests that our study adds new insights into the microbiome composition of these sponges in a tropical marine environment. However, the association of specific Actinomycetes with particular sponge species cannot be conclusively determined at this stage. Further studies are needed to confirm these findings through repeated sampling across geographic locations and during seasons to account for potential temporal and spatial variability. Additionally, environmental factors such as water temperature, salinity, and nutrient availability may play a significant role in shaping the microbiome composition of these sponges, further highlighting the need for broader studies to fully understand the dynamics of sponge-associated Actinomycetes in tropical marine environments.

In other marine environments, such as temperate and polar regions, different genera, including *Streptomyces* and *Salinispora*, are more commonly isolated from sponges [98], [99]. The dominance of *Micromonospora* in this study, particularly as observed in culture-based methods, may reflect the tropical environmental conditions around Sichang Island, such as higher temperatures and salinity, which are known to influence microbial community composition. This finding contrasts with studies conducted in temperate zones, where other genera often dominate the Actinomycetes communities [100]. Our results align with expectations based on previous studies of tropical marine sponges, where *Micromonospora* and *Streptomyces* have been commonly reported. However, the discrepancy between the cultured strains and the sequencing data highlights methodological limitations, particularly the potential underrepresentation of certain Actinomycetes due to primer bias or culture conditions. This emphasizes the need for more comprehensive approaches, including the use of diverse isolation media and Actinomycetes-specific primers, to fully capture the diversity of sponge-associated Actinomycetes. Despite these challenges, the findings underscore the importance of exploring under-sampled environments like the Gulf of Thailand, which may harbor novel Actinomycetes with unique bioactive potential.

## 5. Conclusions

This study highlights the bacterial diversity within tropical marine sponges. The combination of culture-dependent and independent methods reveal distinct microbial communities, with *Micromonospora* being prominent among the isolated Actinomycetes. The findings underscore the biotechnological potential of marine sponges as sources of novel microorganisms, though many remain unculturable, suggesting that further metagenomic research is needed to fully explore their potential. These results offer promising insights for bioprospecting efforts in drug discovery and biotechnology.

## Use of AI tools declaration

The authors declare they have not used Artificial Intelligence (AI) tools in the creation of this article.




