# Supplementary material for: Microbiome profiling and Actinomycetes isolation from tropical marine sponges
Source: AIMS Microbiol. 2025 Mar 3;11(1):182–205. doi: 10.3934/microbiol.2025010 (PMC11950688; doi:10.3934/microbiol.2025010)
Supplement: Supplementary file 1 [file microbiol-11-01-010-s001.pdf]

AIMS Microbiology

AIMS Microbiology, 11(1): 182–205.
DOI: 10.3934/microbiol.2025010
Received: 22 November 2024
Revised: 19 February 2025
Accepted: 26 February 2025
Published: 03 March 2025

https://www.aimspress.com/journal/microbiology

*Research article*

# Microbiome profiling and Actinomycetes isolation from tropical marine sponges

**Trinset Weeraphan[1], Chollabuppha Chou[1], Naphatson Chanthathamrongsiri[2,3], Thanchanok Sirirak[2,3], Sumaitt Putchakarn[4], Supakarn Chamni[5,6] and Wongsakorn Phongsopitanun[1,6,*]**

[1] Department of Biochemistry and Microbiology, Chulalongkorn University, Pathum Wan, Bangkok, 10330, Thailand
[2] Faculty of Pharmaceutical Sciences, Burapha University, Chonburi, 20131, Thailand
[3] The Research Unit in Synthetic Compounds and Synthetic Analogues from Natural Product for Drug Discovery (RSND), Burapha University, Chonburi, 20131, Thailand
[4] Institute of Marine Science, Burapha University, 20131, Thailand
[5] Department of Pharmacognosy and Pharmaceutical Botany, Faculty of Pharmaceutical Sciences, Chulalongkorn University, Bangkok, 10330, Thailand
[6] Center of Excellence in Natural Products and Nanoparticles, Chulalongkorn University, Bangkok, Thailand

* **Correspondence:** Email: wongsakorn.p@chula.ac.th.

## Supplementary

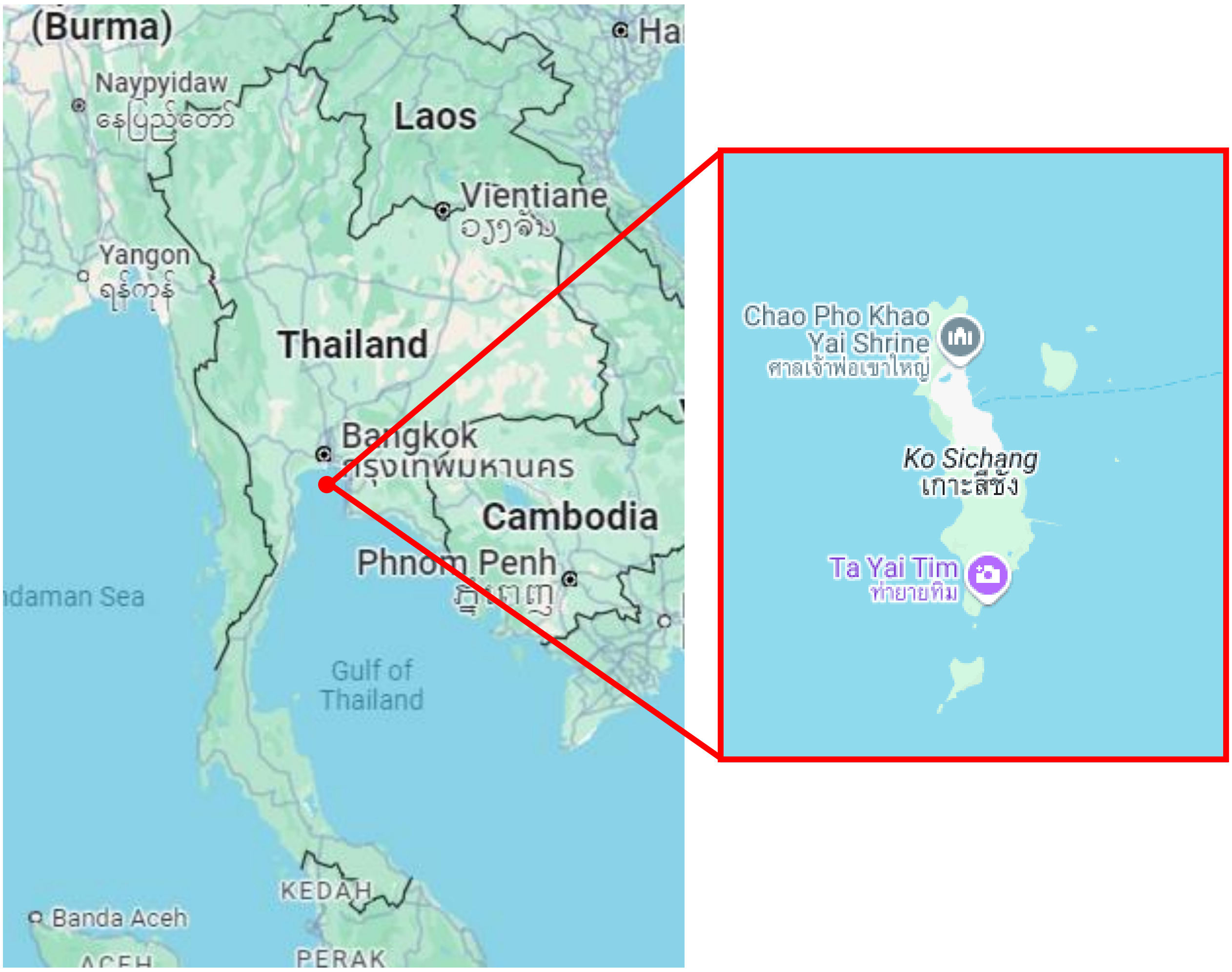


**Figure S1.** Sampling site location (Sichang Island, Gulf of Thailand: 13°08'59.5"N, 100°48'34.5"E).

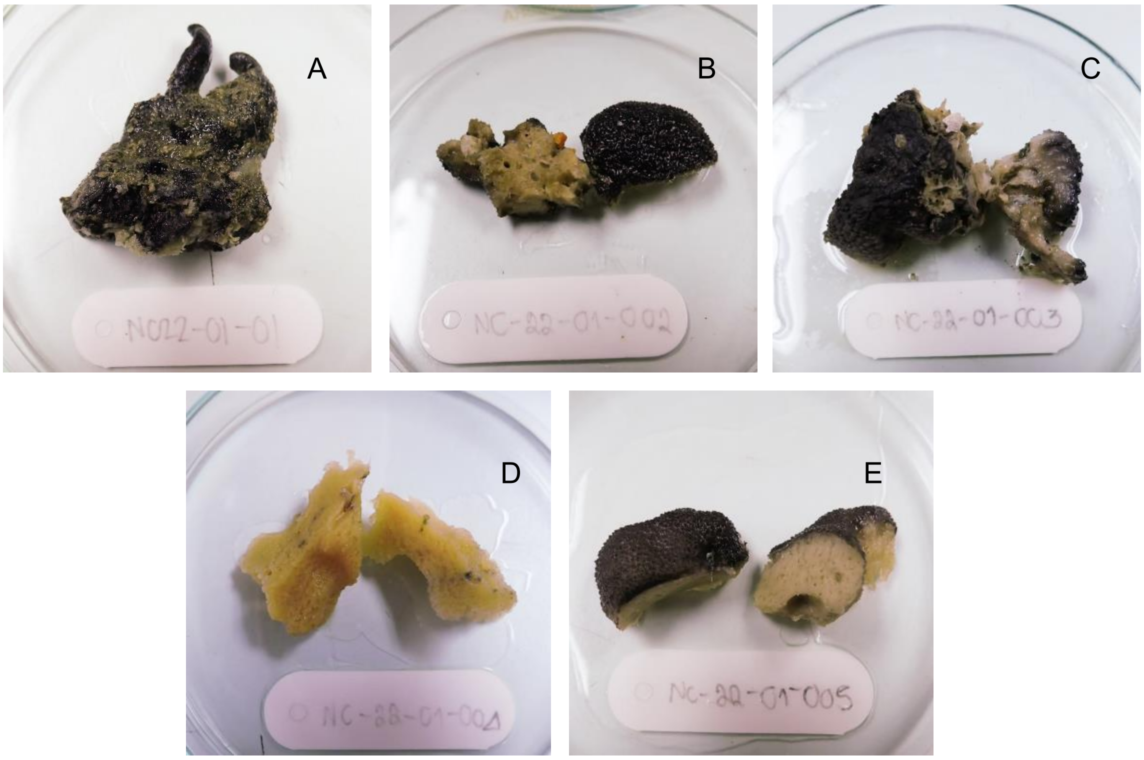


**Figure S2.** Photo of sponge sample used in this study. (A) *Penares nux* (de Laubenfels, 1954) (PF01), (B) *Cacospongia* sp. (PF02), (C) *Ircinia mutans* (Wilson, 1925) (PF03), (D) *Gelliodes petrosioides* (Dendy, 1905) (PF04), (E) *Cacospongia* sp. (PF05).

**Table S1.** Actinobacteria isolated from sponge samples using cultural dependent method.

| Sponge sample | Actinobacterial Isolate no. | The closest type strains (Valid name) | Accession number of 16S sequence | Similarity (%) | Length (bp) |
|---|---|---|---|---|---|
| PF1 (*Penares nux* (de Laubenfels, 1954)) | PF1-02 | *Streptomyces iranensis* HM 35$^T$ | PQ361752 | 99.44 | 1,465 |
| | PF1-03 | *Micromonospora humi* DSM 45647$^T$ | PQ361753 | 99.86 | 1,450 |
| | PF1-04 | *Micromonospora endophytica* 202201$^T$ | PQ361754 | 99.78 | 1,369 |
| PF2 (*Cacospongia* sp.) | PF2-02 | *Micromonospora krabiensis* DSM 45344$^T$ | PQ361755 | 100 | 1,445 |
| | PF2-03 | *Micromonospora wenchangensis* CCTCC AA 2012002$^T$ | PQ361756 | 100 | 1,459 |
| | PF2-04 | *Micromonospora fluminis* A38$^T$ | PQ361757 | 100 | 1,453 |
| | PF2-06 | *Micromonospora haikouensis* 232617$^T$ | PQ361758 | 100 | 1,458 |
| | PF2-07 | *Micromonospora fluminis* A38$^T$ | PQ361759 | 99.62 | 1,306 |
| | PF2-08 | *Micromonospora maritima* D10-9-5$^T$ | PQ361760 | 100 | 1,360 |
| | PF2-09 | *Micromonospora maritima* D10-9-5$^T$ | PQ361761 | 99.85 | 1,355 |
| | PF2-10 | *Micromonospora aurantiaca* ATCC 27029$^T$ | PQ361762 | 100 | 1,365 |
| | PF2-11 | *Micromonospora craniellae* LHW63014$^T$ | PQ361763 | 99.64 | 1,381 |
| | PF2-12 | *Micromonospora schwarzwaldensis* HKI0641$^T$ | PQ361764 | 99.42 | 1,369 |
| | PF2-13 | *Micromonospora aurantiaca* ATCC 27029$^T$ | PQ361765 | 100 | 1,337 |
| | PF2-15 | *Micromonospora endophytica* 202201$^T$ | PQ361766 | 99.78 | 1,367 |
| | PF2-16 | *Micromonospora coxensis* DSM 45161$^T$ | PQ361767 | 99.41 | 1,356 |
| | PF2-19 | *Micromonospora chalcea* DSM 43026$^T$ | PQ361768 | 99.78 | 1,347 |
| | PF2-20 | *Micromonospora chalcea* DSM 43026$^T$ | PQ361769 | 99.78 | 1,357 |

*Continued on next page*

| Isolation sample | Isolated | The closest type strains (Valid name) | Accession number of 16S sequence | Similarity (%) | Length (bp) |
|---|---|---|---|---|---|
| PF3 (*Ircinia mutans* (Wilson, 1925)) | PF3-01 | *Streptomyces olivaceus* NRRL B-3009$^{T}$ | PQ361770 | 99.91 | 1,146 |
| | PF3-02 | *Micromonospora fluminis* A38$^{T}$ | PQ361771 | 100 | 1,374 |
| | PF3-03 | *Micromonospora fluminis* A38$^{T}$ | PQ361772 | 100 | 1,321 |
| | PF3-05 | *Micromonospora maritima* D10-9-5$^{T}$ | PQ361773 | 100 | 1,348 |
| | PF3-06 | *Micromonospora radicis*AZ1-13$^{T}$ | PQ361774 | 99.05 | 1,054 |
| | PF3-07 | *Micromonospora maritima* D10-9-5$^{T}$ | PQ361775 | 99.82 | 1,084 |
| | PF3-08 | *Micromonospora fluminis* A38$^{T}$ | PQ361776 | 100 | 1,346 |
| | PF3-10 | *Micromonospora fluminis* A38$^{T}$ | PQ361777 | 99.92 | 1,284 |
| | PF3-13 | *Micromonospora fluminis* A38$^{T}$ | PQ361778 | 100 | 1,343 |
| | PF3-14 | *Micromonospora maritima* D10-9-5$^{T}$ | PQ361779 | 100 | 1,341 |
| | PF3-15 | *Micromonospora taraxaci* DSM 45885$^{T}$ | PQ361780 | 99.57 | 1,393 |
| | PF3-16 | *Micromonospora schwarzwaldensis* HKI0641$^{T}$ | PQ361781 | 99.92 | 1,321 |
| | PF3-17 | *Micromonospora chokoriensis* DSM 45160$^{T}$ | PQ361782 | 100 | 1,347 |
| | PF3-18 | *Micromonospora oryzae* CP2R9-1$^{T}$ | PQ361783 | 100 | 1,420 |
| | PF3-20 | *Micromonospora taraxaci* DSM 45885$^{T}$ | PQ361784 | 99.57 | 1,397 |
| | PF3-22 | *Streptomyces iranensis* HM 35$^{T}$ | PQ361785 | 99.56 | 1,356 |
| | PF3-23 | *Micromonospora fluminis* A38$^{T}$ | PQ361786 | 100 | 1,355 |
| | PF3-24 | *Micromonospora fluminis* A38$^{T}$ | PQ361787 | 100 | 1,352 |
| | PF3-25 | *Micromonospora fluminis* A38$^{T}$ | PQ361788 | 100 | 1,290 |

*Continued on next page*

| Isolation sample | Isolated | The closest type strains (Valid name) | Accession number of 16S sequence | Similarity (%) | Length (bp) |
|---|---|---|---|---|---|
| PF4 (*Gelliodes petrosioides* Dendy, 1905) | PF4-01 | *Micromonospora schwarzwaldensis* HKI0641$^T$ | PQ361789 | 99.4 | 1,344 |
| | PF4-02 | *Micromonospora endophytica* 202201$^T$ | PQ361790 | 99.78 | 1,353 |
| | PF4-03 | *Micromonospora carbonacea* DSM 43168$^T$ | PQ361791 | 99.85 | 1,348 |
| | PF4-04 | *Micromonospora fluminis* A38$^T$ | PQ361792 | 100 | 1,356 |
| | PF4-05 | *Micromonospora wenchangensis* CCTCC AA 2012002$^T$ | PQ361793 | 100 | 1,132 |
| PF5 (*Cacospongia* sp.) | PF5-01 | *Micromonospora haikouensis* 232617$^T$ | PQ361794 | 100 | 1,329 |
| | PF5-03 | *Micromonospora oryzae* CP2R9-1$^T$ | PQ361795 | 99.85 | 1,352 |
| | PF5-05 | *Micromonospora schwarzwaldensis* HKI0641$^T$ | PQ361796 | 99.4 | 1,340 |
| | PF5-06 | *Micromonospora wenchangensis* CCTCC AA 2012002$^T$ | PQ361797 | 99.48 | 1,347 |
| | PF5-08 | *Micromonospora maritima* D10-9-5$^T$ | PQ361798 | 100 | 1,237 |
| | PF5-09 | *Micromonospora schwarzwaldensis* HKI0641$^T$ | PQ361799 | 99.4 | 1,328 |

AIMS Press

© 2025 the Author(s), licensee AIMS Press. This is an open access article distributed under the terms of the Creative Commons Attribution License (https://creativecommons.org/licenses/by/4.0)
